# Removal of Metals from the System by Galvanism

**Published:** 1855-08

**Authors:** G. Huff

**Affiliations:** Lexington, Kentucky


*Removal of Metals from the System by Galvanism.* By G. Huff, M. D., Lexington, Kentucky.

To the Editors of the New York Medical Times :

Gentlemen—Having experienced the beneficial effects of galvanism in extracting metallic poisons from the human organism, and believing it to be a subject of much interest to the profession, I now place at your disposal a report of my experiments.

Very respectfully yours, G. Huff.

Case I.—Mrs. W——, aged 27 years, of lymphatic temperament, with auburn hair and white skin, had been under treatment for diseased spine fifteen months. During this time she had taken very large quantities of mercury, which, her subsequent medical attendant stated, produced paralysis of the lower extremities.

I was called in consultation by the advice of her physicians, and it was decided that she should be put under treatment by galvanism. Her physicians having thrown upon me the entire responsibility of the case, I took charge of her; and one day, while making an application of this potent agent to the spine, the feet having been placed in a metallic bathing-tub with acidulated water, her husband suddenly called my attention, exclaiming at the same time, "See the mercury!" On making an examination, I found several globules of metallic mercury lying on the bottom of the tub. I continued this (electric) treatment a long time, and she ultimately recovered, and now enjoys the powers of locomotion most perfectly.

Case II.—Mr. B——, aged 40 years, of nervous temperament, with dark hair, white and thin skin, had been treated for syphilis for a long period, and had been repeatedly salivated, from which he had suffered severely in the joints. The capsular ligament was so much elongated as to cause luxation of the head of the *femur*; separation of the *carpus* of each hand had taken place, and the metacarpal joints of the fingers were very much enlarged. He had been under treatment of physicians who stood deservedly high in the profession, and had visited warm springs in Arkansas by their advice. At this time he could scarcely move with crutches, even with the help of two attendants which he took with him. He remained there one winter, and returned without having obtained any relief. His friends then advised him to apply to me, and, with the consent of his physicians, he did so. On examination of his case I concluded to treat him, and commenced with warm baths, which invariably left him worse, the joints becoming more stiff and painful, and with less mobility. Believing that the remote cause of this aggravation of his disease was the presence of mercury in his system, I was induced to attempt to extract it. To accomplish this, after having placed him in a porcelain bathing-foot tub, with acidulated water, and a metallic plate beneath his feet, I completed the circuit, and after the lapse of twenty minutes I discovered a light-white precipitate, and the impress of his toes left on the plate of a light bluish color, with silvery lustre. I repeated this operation several times, and then commenced the galvanic treatment for rheumatism, and infused iodine into the joints in order to produce absorption of abnormal secretion that had formed there. From this time he commenced to improve, and went on improving without a relapse. All the joints have now recovered their normal condition, with the exception of the left hip-joint, the femur of that side now remaining seven-eighths of an inch below the right, although it has ascended three-eighths of an inch during my treatment; his general health has very much improved; in fact, he says it is now as good as it has been at any period of his life.

Case III. Mrs. N——, aged 28 years, of bilious temperament, small size, hair and eyes black, of a very high order of intellect. At the birth of her second child there was very profuse hæmorrhage, and much inflammation of the uterus was superadded in consequence of medicines having been imprudently given by her physicians to facilitate labor. For the purpose of suppressing the hæmorrhage and restoring the uterus to a healthy condition, sugar of lead was given in small doses, and its use continued a long time. This treatment resulted in *lead palsy* (the total loss of muscular contraction of the extremities.) In order to extract the lead from her system, I commenced the treatment by galvanism in the same manner as in the foregoing case, and with the same results, except that the precipitate was of a dark grey color, and the impress of the toes left on the plate was of a darker hue. When the paralysis was nearly removed, partial amaurosis set in, and ultimately became total. I treated this without benefit, although I think the treatment has not been fully tested, as she was obliged to return home, in consequence of domestic cares, sooner than I anticipated.

*Lexington, Ky., May 8th,* 1855.

[The preceding communication contains results which will doubtless be novel to most of our readers, and which would seem to promise to be of value in a therapeutic point of view. It derives additional interest from the fact that, at the meeting of the Imperial Academy of Medicine of Paris, held January 29, 1855, a note was presented from MM. Verguès and A. Poey, on *the new application of electro-chemistry to the removal of metals from the system.* We extract the following account of the disease and process from the *L'Abeille Medicale,* 15th Feb., 1855:—

M. Vergués, who had on the back of his hand an ill-conditioned ulcer, caused by the introduction of metallic substances in the process of gilding and silvering by galvanism, on plunging his hands into an electro-chemical bath at the positive pole, found to his great surprise, a metallic plate in contact with the negative pole, covered, at the end of a quarter of an hour, with a thin layer of gold and silver. A few baths proved sufficient to radically cure the ulcers, which had previously resisted the most active means. The first experiment was made at New York, the 16th of April, 1852, and was followed by several others, which had led to the introduction of a new therapeutic mode of removing metals from the system.

The patient is placed up to the neck in a metallic bathing tub, isolated from the ground, and made to rest in a horizontal position upon a wooden bench, the whole length of the body, which is to be also isolated from the bathing tub. The water is to be acidulated with the nitric or the hydrochloric acid for the removal of mercury, gold, and silver, and with sulphuric acid for the removal of lead.

One extremity of the bath is put in contact with the negative pole of the pile by means of a screw, and the patient takes hold of the positive pole sometimes with the right hand and sometimes with the left. Tho arm is held up by supports in contact with the seat. The extremity of the positive conductor which the patient holds is armed with a massive iron handle, wrapped around with linen, to diminish the calorific action of the current, which is very powerful, and which, without this prevention would burn the hands.

The patient being thus placed, the positive current enters either by the right or left arm, circulates from the head to the feet, and is neutralized at the negative pole, on the sides of the bathing tub. Being isolated from direct contact with the negative pole as well as from the ground, the electric fluid radiates from the body into the bath, forming a multitude of currents from the entire surface of the body, which, after having traversed the internal organs and even the bones, neutralize themselves upon the negative side of the bathing tub.

They say that they have thus withdrawn from the femur and tibia of a patient a large quantity of mercury, which, according to the opinion of several physicians, had remained there fifteen years.

The paper was referred to a commission, consisting of MM. Dumas, Rayer, and Cl. Bernard.

The *Virginia Med. and Surgical Journal*, for May 1, 1855, contains also (in addition to the above) an account of an experiment made before the members of the Faculty of Medicine of Havana, in which a similar result followed the use of the same agent.

The metallic spots formed by this process are said to vary in size from that of the head of a pin to the size of a pea, while some are microscopic.

In a letter received from Dr. Huff, subsequently to his communication, he says that he had never seen the process, nor read any work respecting what he calls "his method of extracting metals from the human system." He says, "my mode is constantly demonstrated by the ordinary course pursued for the electrolysis of metallic salts, by those engaged in electrotyping and electroplating." He speaks also of the solution of urinary deposits in the bladder by galvanism without any *difficulty or pain*, and promises to communicate some interesting results on the subject.

The report of the Imperial Cabinet of Medicine has been copied in different journals in our country, and has been hailed as embodying a valuable contribution to therapeutics; and if future results confirm the hopes thus entertained, the paper of Dr. Huff will be invested with additional interest.—*Eds. N. Y. Med. Times.*

